# COVID-19 Surveillance and Investigations in Workplaces — Seattle & King County, Washington, June 15–November 15, 2020

**DOI:** 10.15585/mmwr.mm7025a3

**Published:** 2021-06-25

**Authors:** Jesse Bonwitt, Ruth W. Deya, Dustin W. Currie, Beth Lipton, Melinda Huntington-Frazier, Sara Jaye Sanford, Aley Joseph Pallickaparambil, Julia Hood, Agam K. Rao, Kaitlin Kelly-Reif, Sara E. Luckhaupt, Sargis Pogosjans, Scott Lindquist, Jeff Duchin, Vance Kawakami, Public Health — Seattle & King County COVID-19 Community Investigation Team, Public Health — Seattle & King County Analytics and Informatics Team, Public Health — Seattle & King County COVID-19 Community Investigation Team, Rosheen Birdie, Siri Bliesner, Erika Bro, Thu Bui, Joan DaCruz, Maryellen Elcock, Joanie Lambert, Ming Leung, Angelo Marfa, Temet Mcmichael, Laura Mummert, Aaron Pomerantz, Nicola Sifrit, Renee Sutton, Christine Thomforde, Christian VonJoe, Public Health — Seattle & King County Analytics and Informatics Team, Jennifer Look, Holly Whitney

**Affiliations:** ^1^CDC COVID-19 Response Team; ^2^Public Health — Seattle & King County, Seattle, Washington; ^3^Epidemic Intelligence Service, CDC; ^4^Washington State Department of Health.; Public Health — Seattle & King County; Public Health — Seattle & King County; Public Health — Seattle & King County; Public Health — Seattle & King County; Public Health — Seattle & King County;; Public Health — Seattle & King County; Public Health — Seattle & King County; Public Health — Seattle & King County; Public Health — Seattle & King County; Public Health — Seattle & King County; Public Health — Seattle & King County; Public Health — Seattle & King County; Public Health — Seattle & King County; Public Health — Seattle & King County; Public Health — Seattle & King County; Public Health — Seattle & King County.; Public Health — Seattle & King County; Public Health — Seattle & King County

Workplace activities involving close contact with coworkers and customers can lead to transmission of SARS-CoV-2, the virus that causes COVID-19 (*1*,*2*). Information on the approach to and effectiveness of COVID-19 workplace investigations is limited. In May 2020, Public Health — Seattle & King County (PHSKC), King County, Washington established a COVID-19 workplace surveillance and response system to enhance COVID-19 contact tracing and identify outbreaks in workplaces. During June 15–November 15, 2020, a total of 2,881 workplaces in King County reported at least one case of COVID-19. Among 1,305 (45.3%) investigated workplaces,* 524 (40.3%) met the definition of a workplace outbreak.^†^ Among 306 (58.4%) workplaces with complete data,^§^ an average of 4.4 employee COVID-19 cases^¶^ (median = three; range = 1–65) were identified per outbreak, with an average attack rate among employees of 17.5%. PHSKC and the Washington State Department of Health optimized resources by establishing a classification scheme to prioritize workplace investigations as high, medium, or low priority based on workplace features observed to be associated with increased COVID-19 spread and workforce features associated with severe disease outcomes. High-priority investigations were significantly more likely than medium- and low-priority investigations to have two or more cases among employees (p<0.001), two or more cases not previously linked to the workplace (p<0.001), or two or more exposed workplace contacts not previously identified during case interviews (p = 0.002). Prioritization of workplace investigations allowed for the allocation of limited resources to effectively conduct workplace investigations to limit the potential workplace spread of COVID-19. Workplace investigations can also serve as an opportunity to provide guidance on preventing workplace exposures to SARS-CoV-2, facilitate access to vaccines, and strengthen collaborations between public health and businesses.

Workplaces that met the investigation threshold during June 15–November 15, 2020 were assessed. Workplaces met the investigation threshold if one or more COVID-19 patients attended work while contagious** or two or more COVID-19 patients from the same workplace reported symptom onset within 14 days (or received a positive SARS-CoV-2 reverse transcription–polymerase chain reaction [RT-PCR] or antigen test result if asymptomatic). Information to determine whether workplaces met the investigation threshold was collected during routine patient interviews and through daily review of a list of workplaces where one or more COVID-19 patients attended work while contagious. 

Workplaces were prioritized as high-, medium-, or low-priority for investigation based on information collected during routine patient interviews. Workplaces meeting at least one of the following criteria were classified as high priority: 1) workplaces with two or more laboratory-confirmed (RT-PCR or antigen test) COVID-19 cases in which symptom onset occurred within 14 days (or asymptomatic workers who received a positive laboratory test result); 2) workplaces with an infected person who mentioned coworkers had received positive test results or had COVID-like symptoms; 3) workplaces with an infected person without phone numbers for exposed coworkers or customers; or 4) workplaces in which at least one person with laboratory-confirmed COVID-19 infection reported going to work while contagious, and one of the following: a) at least five potential close contacts^††^ with other coworkers or customers, b) was an industry with a high number of customers or a high-density workplace,^§§^^,^ c) had a disproportionate number of workers at higher risk for infection or disproportionally affected or restricted populations, or d) had workers with concerns about an absence of infection control measures in the workplace because they worked in close contact with coworkers or customers. Medium-priority workplaces were those in which at least one person with laboratory-confirmed infection reported going to work while contagious and one of the following: a) workers reported working in close contact with coworkers or customers, b) workers had concerns about an absence of infection control measures in the workplace, c) was an industry with a likely high number of customers, or d) was a workplace with a prior documented COVID-19 outbreak or other concerns that were flagged during the case investigation (e.g., patient was not allowed to take COVID-19 leave or was allowed to go to work while contagious). Low-priority workplaces were those with at least one laboratory-confirmed case in which the patient reported going to work while contagious, and other criteria not included for high- and medium-priority workplaces. 

Medium- and low-priority workplaces were only investigated once all high-priority workplaces had been assigned to investigators. A workplace investigation entailed working with occupational health services, human resources, or managers to identify all cases and contacts, assessing workplace adherence to COVID-19 prevention and control guidelines, and responding to outbreaks.^¶¶^ An investigation was closed 14 days after the last identified patient was known to be in the workplace during their contagious period (later revised to 28 days); investigations that could not be initiated within 14 days of notification were not pursued.

During the analysis period, workplace outbreak characteristics were calculated by notification method, industry type,*** and number of on-site employees (<10, 10–49, 50–249, or ≥250). Assessment of outbreak characteristics included number of cases and attack rate among employees (cases among employees divided by total on-site workforce). The effectiveness of workplace investigations was evaluated by assessing 1) the number of exposed workplace contacts identified that had not been elicited during case interviews; 2) the number of identified cases not previously linked to the workplace; and 3) the number of cases among employees. Timeliness was evaluated by examining the interval between notification date and investigation date, and time spent on an investigation. The ratio of notification, investigations, and outbreaks to all community cases occurring in King County during the same period was calculated. Data were collected using the Research Electronic Data Capture (REDCap; version 11.0.3; Vanderbilt University) data management platform (*3*). Descriptive analyses were performed in R (version 3.5.1; R Foundation). Statistically significant differences (p<0.05) by priority level and outbreak status were assessed using Wilcoxon Rank Sum test (medians) and Pearson’s chi-square test or Fisher’s exact test (proportions). This activity was reviewed by CDC and conducted consistent with applicable federal law and CDC policy.^†††^

During June 15–November 15, 2020, a total of 2,881 King County workplaces met the investigation threshold (108 notifications per 1,000 community cases). Among 2,850 workplaces with known priority level, 1,770 (62.1%) were classified as high-, 702 (24.6%) as medium-, and 378 (13.3%) as low-priority investigations. A total of 1,404 (48.7%) workplaces were contacted, 99 (3.4%) of which did not require a full investigation because the employee was determined not to have been at work while infectious. Overall, 1,305 (45.3%) of 2,881 workplaces were investigated (49 investigations per 1,000 community cases) (Table 1). Of 1,300 investigated workplaces with complete information, 524 (40.3%) met the definition of an outbreak (19.6 outbreaks per 1,000 community cases). Among 1,085 completed high-priority investigations, 489 (45.1%) met the definition of an outbreak, compared with 35 of 217 (16.1%) completed medium- and low-priority investigations. Among the 1,477 (51.3%) workplaces not investigated, 1,232 (84%) investigations could not be initiated within 14 days.^§§§^

**TABLE 1:** COVID-19 workplace notifications,* investigations, and outbreaks,**^†^** by priority classification**^§^** — Seattle & King County, Washington, June 15–November 15, 2020

Workplace status	Priority				
	Total	High	Medium	Low	Unknown
Notifications, no.	**2,881**	1,770	702	378	31
Investigated, no. (%)	**1,305 (45.3)**	1,085 (61.3)	191 (27.2)	26 (6.9)	3 (9.7)
Outbreak identified, no. (%)	**524^¶^ (18.2)**	489 (27.6)	28 (4.0)	7 (1.9)	0 (—)

**Abbreviation**: RT-PCR = reverse transcription–polymerase chain reaction.

* Workplaces met the investigation threshold if at least one COVID-19 patient attended work while contagious or two or more COVID-19 patients from the same workplace reported symptom onset within 14 days (or received a positive SARS-CoV-2 RT-PCR or antigen test result if asymptomatic). Period of contagiousness was defined as 2 days before onset of any symptoms (or 2 days before the date of specimen collection for a confirmed laboratory test in asymptomatic persons) through the beginning of isolation.

^†^ A workplace outbreak (cluster) was defined by the Washington State Department of Health as the occurrence of two or more cases of RT-PCR- or antigen-confirmed cases of SARS-CoV-2 infection from the same workplace in which symptom onset occurred within 14 days (or positive laboratory test result if asymptomatic), a plausible epidemiologic link in the workplace, and no known epidemiologic link outside the workplace.

^§^ Priority levels were assigned based on workplace features assumed to be associated with increased COVID-19 spread, and workforce features associated with severe disease outcomes.

^¶^ An outbreak determination was not available for two workplaces.

Among 838 workplaces with complete relevant data, the median interval between symptom onset (or positive laboratory test result for asymptomatic cases) of the first reported case associated with the workplace and notification to PHSKC was 6 days (interquartile range [IQR] = 4–9 days). In these workplaces, 295 (56%) outbreaks were identified during routine case investigations, 124 (24%) outbreaks were self-reported by workplaces (voluntary or mandated), and 106 (20%) outbreaks were identified through other means. Among 306 workplaces with complete data on number of cases, the average outbreak involved 4.4 employees (median = three; range = 1–65), with an average attack rate of 17.5% (median = 8.9%; range = 0.1%–100%), among 287 workplaces with complete attack rate data (Table 2). A total of 1,347 cases were associated with these workplace outbreaks, representing 5.0% of the 26,703 total COVID-19 cases reported in King County during the same period.

**TABLE 2:** COVID-19 workplace notifications,* investigations, outbreaks,**^†^** and outbreak characteristics, by industry type**^§^** and workplace size — Seattle & King County, Washington, June 15–November 15, 2020

Characteristic	Workplace status, no. (column %)			Median (IQR)		
	Notifications	Investigations	Outbreaks	Employee cases^¶^	Outbreak-associated employee cases**	Outbreak-associated employee attack rate^††,§§^
**Industry type**						
Govt. or public administration	39 (1.4)	28 (2.1)	8 (1.5)	1 (1–2)	2 (2–3)	10.4 (6.5–21.9)
Service provision	1,005 (34.9)	707 (54.2)	209 (39.9)	1 (1–2)	3 (2–4)	8.8 (3.1–20)
Goods production	300 (10.4)	203 (15.6)	110 (21.0)	2 (1–4)	4 (2–3)	8.9 (3.3–22.7)
Other	29 (1.0)	27 (2.1)	7 (1.3)	1 (1–2)	3 (2.5–5.5)	10.0 (4.1–20.5)
Unknown	1,508 (52.3)	340 (26.1)	190 (36.3)	1 (1–1.5)	2 (2–2)	9.5 (9.5–9.5)
**Total**	**2,881**	**1,305**	**524**	**1 (1**–**2)**	**3 (2–5)**	**8.9 (3.3–22.1)**
**Workplace size, no. of on-site employees**						
≥250	46 (1.6)	37 (2.8)	21 (4.0)	2.5 (1–6)	3.5 (2.8–6)	1.1 (0.5–2)
50–249	225 (7.8)	327 (25.1)	105 (20.0)	2 (1–4)	3 (2–5)	3.3 (1.9–5.7)
10–49	346 (12.0)	212 (16.2)	110 (21.0)	1 (1–2)	3(2–4)	12.8 (8.7–18.2)
<10	196 (6.8)	178 (13.6)	52 (9.9)	1 (1–2)	2 (2–4)	40.0 (28.6–66.7)
Unknown	2,068 (71.8)	551 (42.2)	236 (45.0)	1 (1–2)	2 (2–5.5)	—^¶¶^
**Total**	**2,881**	**1,305**	**524**	**1 (1**–**2)**	**3 (2–5)**	**8.9 (3.3–22.1)**

**Abbreviation:** IQR = interquartile range.

* Workplaces met the investigation threshold if one or more COVID-19 patients attended work while contagious or two or more COVID-19 patients from the same workplace reported symptom onset (or a positive SARS-CoV-2 RT-PCR or antigen test result if asymptomatic) within 14 days. Period of contagiousness was defined as 2 days before onset of any symptoms (or 2 days before the date of specimen collection for a confirmed laboratory test in asymptomatic persons) through the beginning of isolation.

^†^ A workplace outbreak (cluster) was defined by the Washington State Department of Health as the occurrence of two or more cases of reverse transcription–polymerase chain reaction (RT-PCR)- or antigen-confirmed cases of SARS-CoV-2 infection from the same workplace with symptom onset within 14 days (or positive laboratory test result if asymptomatic), a plausible epidemiologic link in the workplace, and no known epidemiologic link outside the workplace.

^§^ Industry types described in this report included the following: 1) government agency or facility (e.g., military and public safety); 2) service provision (e.g., food service and restaurants, recreation and hospitality, personal care, retail, and transportation); and 3) goods production (e.g., agriculture, produce packing, construction, food and food-related manufacturing, non-food manufacturing).

^¶^ Total of 813 workplaces with completed investigation.

** Total of 306 workplaces with completed investigation and confirmed outbreak.

^††^ Total of 287 workplaces with completed investigation and confirmed outbreak.

^§§^ Number of cases among employees divided by total on-site workforce.

^¶¶^ Attack rate could not be calculated as denominator was unknown.

The median interval between notification and investigation (among 1,142 workplaces with complete data on this metric) was 2 days (IQR = 1–5 days) and was significantly lower in high-priority investigations (2 days; IQR = 1–5 days) than in medium- and low-priority investigations (3 days; IQR = 1–8 days) (Table 3). The median time (minutes) spent per investigation was significantly higher in high-priority investigations (60 minutes; IQR = 45–100 minutes) than in medium- and low-priority investigations (50 minutes; IQR = 30–60 minutes). Among 191 workplaces with complete information on contacts, workplace investigation uncovered an average of 2.7 contacts not previously elicited (median = one; range = 1–35). Among 507 workplaces with complete information on cases, an average of 0.5 cases not previously linked to the workplace (median = 0; range = 0–11) were identified. High-priority investigations were more likely than were medium- and low-priority investigations to identify two or more exposed workplace contacts not previously elicited, two or more cases not previously linked to the workplace, or two or more employee cases. These metrics were also significantly higher (p≤0.001) in outbreaks than in investigations that did not meet the definition of a workplace outbreak.^¶¶¶^

**TABLE 3:** Timeliness and effectiveness of workplace investigations, by workplace prioritization* — Seattle & King County, Washington, June 15–November 15, 2020

Features	Priority, no. (row %)			p-value^†^
	Total	High	Medium and low	
**Timeliness**				
Interval between notification and investigation, days, median (IQR)^§^	2 (1–5)	2 (1–5)	3 (1–8)	0.002
Duration spent on an investigation, minutes, median (IQR)^¶^	60 (40–90)	60 (45–100)	50 (30–60)	<0.001
**Effectiveness**				
**Exposed contacts not previously elicited during patient interviews**				
0–1	96	60 (62.5)	36 (37.5)	0.002
≥2	95	79 (83.2)	16 (16.8)	
**Identified employee cases not previously linked to the workplace**				
0–1	452	335 (74.1)	117 (25.9)	0. 001
≥2	55	52 (94.5)	3 (5.5)	
**No. employee cases identified**				
0–1	450	350 (77.8)	100 (22.2)	<0.001
≥2	363	337 (92.8)	26 (7.2)	

**Abbreviation**: IQR = interquartile range.

* Priority levels were assigned based on workplace features observed to be associated with increased COVID-19 spread, and workforce features associated with severe disease outcomes.

^†^ P-value comparisons using Wilcoxon Rank-Sum test to compare medians and Pearson’s chi-square test or Fisher’s exact test to compare categorical data. Number of workplaces varies by metric because of incomplete data.

^§^ Total of 1,142 workplaces.

^¶^ Total of 671 workplaces.

## Discussion

These King County COVID-19 workplace investigations identified contacts not previously elicited and cases not previously linked to the workplace. The difficulty in eliciting contacts has been documented (*4*) and might be particularly challenging in workplaces where employees might be unable or reluctant to name close contact coworkers. Conversely, workplace outbreaks were primarily identified during case interviews rather than through self-report by employees or businesses, demonstrating the importance of conducting workplace investigations in addition to case interviews and the utility of eliciting detailed workplace information during case interviews.

Given the substantial volume and time-intensive nature of workplace investigations, a prioritization scheme could maximize investigation effectiveness in identifying close contacts, cases, and outbreaks (*5*); CDC has issued similar guidance on prioritization of case investigation and contact tracing (*6*). Improved understanding of occupational risk factors for SARS-CoV-2 infection in workplaces could be used to further refine prioritization (*7*), thereby reducing community transmission through rapid isolation and quarantine of workplace-associated cases and contacts.

Effectiveness metrics were higher in workplace outbreaks than in investigations not meeting the definition of an outbreak. Although this is expected given that workplace outbreaks will generate more cases and contacts, it suggests that prioritizing only workplaces with two or more cases (i.e., those most likely to be outbreaks) could be more efficient. However, this approach could risk missing outbreaks; in this analysis, not all cases were linked to a workplace during the initial interview, and less than one quarter of businesses with outbreaks self-reported to PHSKC.

The findings in this report are subject to at least four limitations. First, only one half of workplaces were investigated, only a small proportion of which were categorized as low-priority for immediate investigation, which could have biased the results toward increased effectiveness of investigating high-priority workplaces. Second, for a high proportion of workplaces, effectiveness data were missing, which could have resulted in bias if the lack of effectiveness data was related to both effectiveness and priority classification. Third, misclassification of workplace exposures and outbreaks might have occurred because of the challenge in ascertaining epidemiologic links when cases have multiple high-risk exposures (e.g., workplace and community exposures). Finally, whereas the number of cases associated with workplace outbreaks as a proportion of the total number of cases in King County (5.0%) was similar to that reported in Wisconsin (5.2%) (*8*), it was less than that reported in Utah (12%) (*9*), suggesting potential underreporting of workplaces-associated cases.

Workplace investigations can enhance the effectiveness of contact tracing and identification of workplace outbreaks, which can inform the implementation of strategies to prevent the spread of COVID-19. Prioritizing workplace investigations based on workplace and workforce characteristics gathered during patient interviews can optimize investigation timeliness and effectiveness in resource-constrained settings (*5*,*10*). Workplace investigations can also serve as an opportunity to provide guidance on preventing workplace exposures to SARS-CoV-2 (*1*), facilitate access to vaccines, and strengthen collaborations between public health and businesses.

**Summary**

**What is already known about this topic?**

Workplace activities that involve close contact with coworkers and customers can lead to COVID-19 spread.

**What is added by this report?**

Workplace investigations were prioritized using workplace features associated with increased COVID-19 spread and with severe disease outcomes. High-priority investigations were more likely than were medium- and low-priority investigations to have two or more cases among employees, two or more cases not previously linked to the workplace, or two or more exposed workplace contacts not previously elicited during case interviews.

**What are the implications for public health practice?**

Workplace investigations uncovered contacts not previously elicited and cases not previously linked to the workplace, demonstrating the importance of conducting workplace investigations in addition to routine case interviews to limit the potential workplace spread of COVID-19.

## Footnotes

* Workplaces related to health care, education, child care, correctional facilities, and congregate living settings are managed separately by PHSKC and are not included in this report.

† A workplace outbreak (cluster) was defined by the Washington State Department of Health as the occurrence of two or more cases of reverse transcription–polymerase chain reaction (RT-PCR) or antigen-confirmed cases of SARS-CoV-2 infection among patients from the same workplace with symptom onset within 14 days (or positive laboratory test result if asymptomatic), a plausible epidemiologic link in the workplace, and no known epidemiologic link outside the workplace.

§ Fifteen workplaces reported only one case among employees but were classified as workplace outbreaks because multiple cases among customers were identified. Among 261 workplace outbreaks with complete information, 32 customers were linked to workplace outbreaks, but were not included in this report.

¶ A COVID-19 case was defined as a positive SARS-CoV-2 RT-PCR or antigen test result.

** Period of contagiousness was defined as 2 days before onset of any symptoms (or 2 days before the date of specimen collection for a confirmed laboratory test in asymptomatic persons) through the beginning of isolation.

†† A close contact was defined as a person who has been within 6 feet of a contagious person (laboratory-confirmed or a clinically compatible illness) for a cumulative total of ≥15 minutes over a 24-hour period.

§§ A high-density workplace was defined as workplace in which workers were in the workplace for long time periods (e.g., for 8–12 hours per shift), and had prolonged close contact with coworkers. These workplaces included agriculture or produce-packing, construction, fishing vessels, manufacturing (food and food-related), and manufacturing (non-food).

¶¶ Cases were identified by defining the employee population at risk and comparing cases within the population with the list of reported cases, and by contact tracing for known cases. Adherence to COVID-19 prevention and control guidelines was ascertained by assessing workplace policies related to personal protective equipment, engineering controls, and administrative controls.

*** Industry types described in this report included 1) government agency or facility (e.g., military and public safety); 2) service provision (e.g., food service and restaurants, recreation and hospitality, and retail); and 3) goods production (e.g., agriculture, produce packing, construction, and food and non-food manufacturing).

††† 45 C.F.R. part 46.102(l)(2), 21 C.F.R. part 56; 42 U.S.C. Sect. 241(d); 5 U.S.C. Sect. 552a; 44 U.S.C. Sect. 3501 et seq.

§§§ Other reasons for noninvestigation included unavailable contact information (54 [3.6%]), refusal to cooperate (82 [5.5%]), out of jurisdiction (10 [0.7%]), or unknown reason (99 [6.7%]).

¶¶¶ The median number of exposed contacts identified in the workplace who were not previously elicited during patient interviews (in 191 workplaces with complete information) was significantly higher in workplaces with outbreaks (median = 2; IQR = 1–4) than in those without outbreaks (median = 1; IQR = 1–3) (p = 0.001). The median number of employee cases identified who had not previously been linked to the workplace (in 507 workplaces with complete information) was significantly higher in workplaces with outbreaks (median = 1; IQR = 0–2) compared with workplaces without outbreaks (median = 0; IQR = 0–0) (p<0.001). The median number of employee cases identified (in 813 workplaces with complete information) was significantly higher in workplaces with outbreaks (median = 3; IQR = 2–5) compared with workplaces without outbreaks (median = 1; IQR = 1–1) (p<0.001).
